# Prenatal Opioid Exposure and ADHD Childhood Symptoms: A Meta-Analysis

**DOI:** 10.3390/children8020106

**Published:** 2021-02-04

**Authors:** Ashlyn N. Schwartz, Lucia M. Reyes, Laurie L. Meschke, Kristina W. Kintziger

**Affiliations:** 1 The Department of Public Health, The University of Tennessee, Knoxville, TN 37996, USA; llmeschke@utk.edu (L.L.M.); kkintzig@utk.edu (K.W.K.); 2 The Department of Child and Family Studies, The University of Tennessee, Knoxville, TN 37996, USA; luciagmiranda@gmail.com

**Keywords:** prenatal opioid exposure, attention deficit hyperactivity disorder, neonatal abstinence syndrome, neonatal opioid withdrawal syndrome

## Abstract

To systematically investigate the association between prenatal opioid exposure (POE) and attention-deficit hyperactivity disorder (ADHD) symptoms in children 2–18 years old, studies were searched using PubMed, CINAHL, PsycINFO, and Web of Science from January of 1950 to October of 2019. Inclusion criteria were observational studies reporting ADHD symptoms of children with POE compared with non-exposed children or normative data. The study protocol was registered with PROSPERO: CRD42018115967. Two independent reviewers extracted data on hyperactivity/impulsivity, inattention symptoms, ADHD combined subscale symptoms, and sample characteristics. Of 223 articles screened, seven met the inclusion criteria. Data represent 319 children with POE and 1308 non-exposed children from 4.3 to 11.2 mean years from five countries. POE was positively associated with childhood hyperactivity/impulsivity (d = 1.40; 95% CI, 0.49–2.31; *p* = 0.003), inattention (d = 1.35; 95% CI, 0.69–2.01; *p* < 0.0001), and combined ADHD symptoms scores (d = 1.27; 95% CI = 0.79–1.75; *p* < 0.0001). POE was positively associated with ADHD combined symptom scores at preschool (d = 0.83, 95% CI, 0.57, 1.09; *p* < 0.0001) and school age (d = 1.45, 95% CI, 0.85 to 2.04; *p* < 0.0001). Results suggest increased risk of ADHD symptoms during school age. Future research is needed to clarify the relationship between biological, social, and environmental risk and ADHD symptoms for children who experienced POE.

## 1. Introduction

Opioid use amongst reproductive-age women is increasing globally [1,2] and has been associated with infant risk, including neonatal abstinence syndrome (NAS) [3,4,5,6], low birth weight [7], preterm birth [8], and altered neonatal brain development [9]. Consequently, there has been a five-fold increase in NAS within the last two decades [3]. Infants with NAS burden the healthcare system, with longer length of hospital stay and increased costs [10]. Pregnant women with opioid dependency represent a vulnerable group of women who have a history of family socioeconomic adversity, child custody loss, and many psychiatric, social, and obstetric needs [11,12]. Thus, children with prenatal opioid exposure (POE) face disproportionate risks. In addition to the neonatal concerns related to POE, some evidence indicates that POE may confer neurodevelopmental risk that endures beyond infancy. A meta-analysis of five studies of POE children’s neurobehavior by Baldacchino and colleagues found significant impairments for cognitive, psychomotor, and behavioral outcomes compared to non-exposed controls in infant and preschool children [13]. Furthermore, in a meta-analysis by Monnelly and colleagues, six of seven studies found that children with POE had increased behavioral problems compared to non-exposed controls of children less than 2 years [14].

Most consistently in the literature, POE has been associated with behavioral issues in early childhood, primarily with symptoms of attention-deficit hyperactivity disorder (ADHD) [15,16,17,18,19,20,21,22,23]. For instance, in a cohort of children born in Norway, POE predicted elevated levels of inattention and impulsivity at both 2 and 4.5 years compared to non-exposed peers [16]. Similarly, a longitudinal study in New Zealand revealed that children with POE had increased inattention and hyperactivity during preschool years compared to non-exposed controls [15]. However, confounders associated with increased risk for ADHD (e.g., care disruption and prenatal polydrug exposure) were evident in both studies [15,16], thus limiting conclusions about the unique role of POE on ADHD symptoms. Less is known about whether ADHD symptoms persist into the school-age period. In small cross-sectional studies, POE has been linked with elevated hyperactivity at 8.5 years [22], poorer sustained attention in boys 7–12 years [24], and higher inattention and impulsivity at 8.5 years [21]. Behavioral ranking scores of Swedish children with POE have corresponded to normative ADHD scores, including symptoms of activity, inattention, and combined subscale scores of children, although the sample size was limited to 16 children [17]. Utilizing cutoff scores for ADHD in behavioral scales, a longitudinal study in Israel reported that over half of the children with POE raised in the biological mother’s home had ADHD and a quarter of adopted children with POE had ADHD [20]. These findings also appear to indicate a pathway between POE and ADHD symptoms, where adoptive homes may mitigate but not eliminate long-term risk. In sum, even though the cognitive and motor outcomes of older children with POE have been assessed [25], no studies have reviewed POE and ADHD associations in older children. 

To fill these gaps in the literature, this meta-analysis aimed to systematically investigate the strength of the association between POE and ADHD symptoms in children, ages 2–18 years. We investigated symptoms of hyperactivity/impulsivity and inattention separately as well as combined and performed subgroup analyses of preschool (4.3–4.5 mean years) and school-age children (5.4–11.2 mean years). We hypothesized that children 2–18 years with POE will have greater mean scores of inattentive, hyperactive/impulsive, and combined symptoms compared to their non-exposed peers.

## 2. Materials and Methods

This meta-analysis was registered with the International Prospective Register of Systematic Reviews (PROSPERO), registration number CRD42018115967 and was conducted using an a priori protocol following the Preferred Reporting Items for Systematic Reviews and Meta-Analyses (PRISMA) guidelines (Appendix A) [26].

To be included in this meta-analysis, studies had to meet the following inclusion criteria: (1) observational study design; (2) all participants with POE (or polydrug exposure including opioids) with a mean age of 2–18 years at follow-up; (3) inclusion of a control or comparison group or use of an established instrument with normative data available; (4) hyperactive/impulsive and/or inattentive behaviors reported via caregiver or teacher ratings, and (5) sufficient statistical information reported or provided upon contacting lead authors. There were no geographical restrictions, and language was restricted to English.

A literature search was conducted independently by the first and second authors for observational studies of ADHD symptoms in children aged 2–18 years with POE, published between January 1950 and October 2019 by searching the following electronic databases: CINAHL, PubMed, PsycINFO, and Web of Science. Filters for the search included limiting articles to human studies and the English language. The finalized search was run on October 20, 2019 and an additional search was re-run before the final analyses to look for additional studies for inclusion. The following keywords were used: ((opioid OR opiate OR heroin OR methadone OR buprenorphine OR NAS OR MAT OR suboxone OR infant withdrawal syndrome OR Subutex OR OMT OR fentanyl OR morphine OR tramadol OR codeine OR hydrocodone OR oxycodone) AND (in utero OR prenatal) AND (infan* OR toddler OR child* OR adolescen*) AND (ADHD OR hyperactivity OR impulsivity OR attention OR ADD OR neurobehavior OR executive function)).

A standardized data extraction tool provided the following information when available: child impulsivity/hyperactivity, inattention, and combined ADHD subscale scores, outcome assessment mean age, number of participants, type of opioid exposure, polydrug exposure, method to identify opioid exposure, NAS diagnosis, care disruption, socio-economic status, measurement instrument used, respondent of instrument, and country of study. Means and standard deviations were extracted on primary outcome measures.

A quantitative synthesis for eligible studies is included using aggregate data with the Comprehensive Meta-Analysis (CMA) Software (V.3). Means and standard deviations were transformed to standardized scores and mean effect sizes (SMD). An effect size was considered small (0.3–0.4), moderate (0.5–0.7), or large (>0.8) according to Cohen’s d [27]. Assessments for heterogeneity between studies in effect measures were conducted using Higgins I^2^, where I^2^ values greater than 50% were considered significant heterogeneity [27]. Publication bias was visually assessed by reviewing funnel plots for asymmetry and with the Egger test. As eligible studies had large heterogeneity, a random-effect meta-analysis with standardized mean differences were calculated for reported hyperactivity/impulsivity, inattention, and combined ADHD subscale symptoms. Subgroup analyses for age (preschool, 4.3–4.5 mean years; school age, 5.4–11.2 mean years) and control/comparison group (healthy control/comparison, healthy children from mothers without opioid use disorder (OUD); environmental control/comparison, children raised by a father with OUD or children raised in a low socioeconomic status with environmental deprivation or neglect) were conducted for reported combined ADHD subscale symptoms using a random-effect meta-analysis. 

The Newcastle–Ottawa Scale for case control and cohort studies was used to assess the risk of bias in included studies and was rated by two independent reviewers [28]. Disagreements were discussed between the first and second author. The Newcastle–Ottawa Scale has a range of possible scores from 0 to 9, with higher scores indicating a better study quality. A score of 3 or less for a study was considered low quality. A sensitivity analysis was conducted using only studies above this threshold to assess if low quality studies influenced the effect size.

## 3. Results

### 3.1. Study Selection

**Figure 1:**
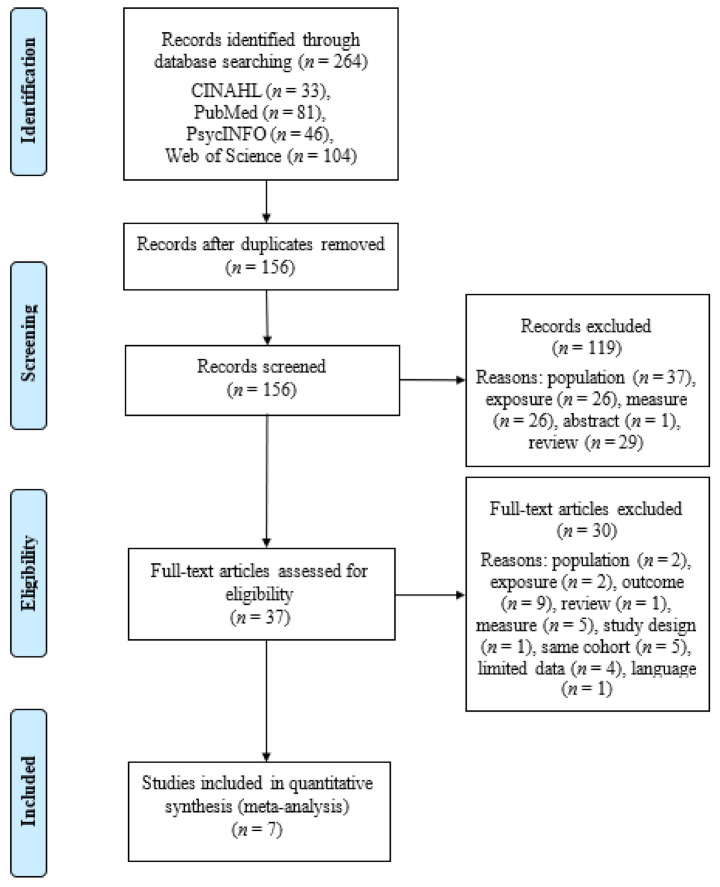
PRISMA flow diagram of literature search.

Overall, 264 articles were included in the literature search. After removal of 108 duplicates, 156 articles were available for title, abstract, and full-text screening. Reasons for article exclusion at each stage are shown in Figure 1. Title and abstract review removed 119 articles and after full-text review, 30 articles were further removed. In total, seven studies were left for inclusion in the meta-analysis. The overall agreement after title and abstract selection was 94% and Cohen’s κ (0.85) and 93% (0.81) at the full text retrieval stage.

Due to the nature of longitudinal follow-up studies, there were five instances where multiple reports were published on different time points of the same cohort [16,18,19,29,30]. In these cases, only one study per primary outcome was included per cohort of children to avoid bias by counting the same participants twice. In such instances, publications with the largest sample sizes, including the highest *n* for primary outcomes, were included in this meta-analysis. When one study published on multiple measures for the same outcome variable, the measure with the largest sample size and with the highest validity and reliability was chosen (*n* = 1) [31]. If a study had more than one control group, the control group whose characteristics most closely represented the non-exposed normative population was chosen (*n* = 1) [20]. Overall, this resulted in excluding five studies in the full-text review stage that met one or more of these exclusion criteria [16,18,19,29,30].

### 3.2. Study Characteristics 

Details regarding characteristics of eligible studies are summarized in Table 1 and Table 2. The seven studies included 319 children with POE and 1308 non-exposed children ranging from 4.2 to 14.2 years of age from Norway [23,31,32], Sweden [17], New Zealand [15], United States [21], and Israel [20]. Studies were published from 1988 to 2018. Polydrug exposure was present in all studies and included the following: tobacco/nicotine, alcohol, marijuana, stimulants, amphetamines, antipsychotic medications, antidepressants, benzodiazepines, opioids/narcotics, and illicit drugs. The main opioid exposure being heroin in one study [23], methadone and/or buprenorphine in four studies [15,17,31,32], and a combination of heroin and methadone in two studies [20,21]. The method to identify opioid exposure included self-report [15,20,23,32], methadone and hospital records [21,23,31], social records [23], and maternal toxicology and infant meconium [15,17]. A change in the primary caregiver, defined as care disruption, was present in all but one study [21], ranging from 14.3% to 98%. In three studies [15,23,32], SES significantly differed between the exposed and non-exposed children. NAS was reported in five of the seven studies [15,17,23,31,32], with reported rates ranging from 17.8% to 88.2%.

Overall, three cohort studies were rated with 5.6 mean quality, ranging from 3 to 7, and four case-control studies were rated 4.3 mean quality, ranging from 2 to 6 (see Table 3). Case control studies of low quality typically had less comparability of cases and controls due to study design or analysis, lower quality ascertainment of POE, different methods of ascertainment from cases and controls, or a different non-response rate [17,31]. The lower quality cohort study had poor comparability of cohorts based on the design or analysis and low-quality assessment of outcome [20].

### 3.3. Behavioral Instruments

The following instruments and subscales were used by the seven studies: the Strengths and Difficulties Questionnaire [hyperactivity/inattention subscale] [34], the Behavior Rating Inventory of Executive Function Preschool version [inhibit and working memory subscales] [35], Swanson Nolan and Pelham Questionnaire [hyperactivity/impulsivity items, and inattention items] [36], Achenbach Child Behavior Checklist [attention scale] [37], Burk’s Behavior Rating Scales [attention and impulse control] [38], and Conner’s Rating Scales for ADHD [combined symptoms] [39]. All behavioral scales included in this meta-analysis are rating scales commonly used in an ADHD diagnosis or measure symptoms of an ADHD diagnosis [40]. All instruments used in analyses were caregiver report, with the exception of one exclusive teacher report on the Strengths and Difficulties Questionnaire [17].

### 3.4. Hyperactivity/Impulsivity Symptoms

**Figure 2:**
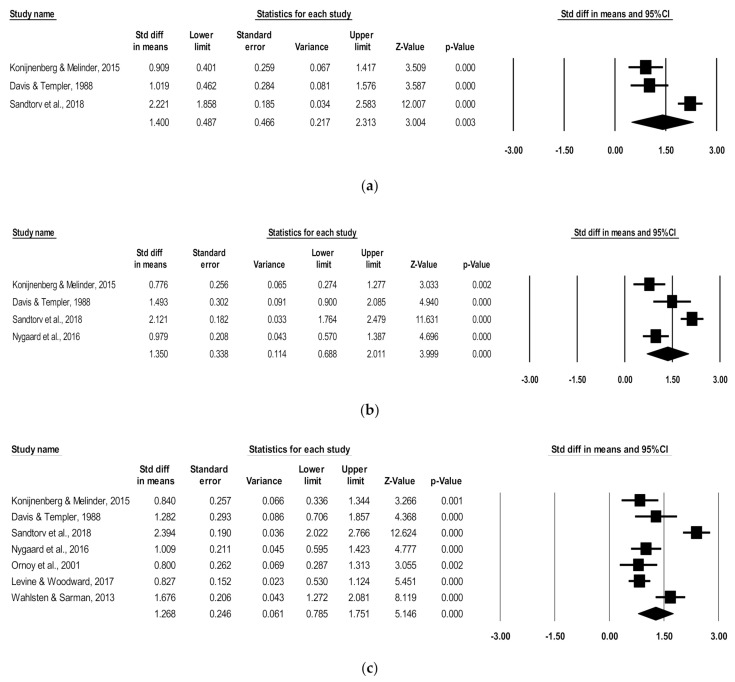
Forest plots comparing (**a**) hyperactivity/impulsivity, (**b**) inattention, and (**c**) combined ADHD subscales of children with POE versus non-exposed children.

Three of the seven studies contained data specific to hyperactivity/impulsivity symptoms and were eligible for individual ADHD symptoms analysis. Thus, three studies were pooled to include 120 children with POE and 230 non-exposed children aging from 4.3 to 11.2 mean years. There was a significant positive association of POE with reported hyperactivity/impulsivity symptoms in childhood (d = 1.40; 95% CI, 0.49–2.31; *p* < 0.003; Figure 2). High heterogeneity was indicated (I^2^ = 91.13); however, the number of studies was too small to perform a subgroup analysis. No studies rated as poor quality met inclusion for this analysis, indicated by scores above 3 on the Newcastle–Ottawa Scale.

### 3.5. Inattention Symptoms 

Of the seven studies, four had data specific to inattention symptoms and were eligible for individual ADHD symptom analysis. These four studies were pooled to include 177 children with POE and 277 non-exposed children ranging in age from 4.3 to 11.2 mean years. There was a significant positive association of POE with reported inattention symptoms in childhood (d = 1.35; 95% CI, 0.69–2.01; *p* < 0.0001) (Figure 2). While high heterogeneity was present (I^2^ = 88.30), the number of included studies was not sufficient to perform a subgroup analysis. No studies rated as poor quality met inclusion for this analysis, which was indicated by scores above 3 on the Newcastle–Ottawa Scale.

### 3.6. Combined Subscale Symptoms

A total of seven studies were pooled to include 319 children with POE and 1308 non-exposed children ranging in age from 4.3 to 11.2 mean years. There was a significant positive association of POE with reported combined ADHD subscale symptoms in childhood (d = 1.27; 95% CI, 0.79–1.75; *p* < 0.0001) (Figure 2). Heterogeneity analysis indicated high variation between studies (I^2^ = 90.54). When removing studies of poor quality [17,20], the significant positive association persisted (d = 1.27, 95% CI, 0.63–1.91; *p* < 0.0001).

**Figure 3:**
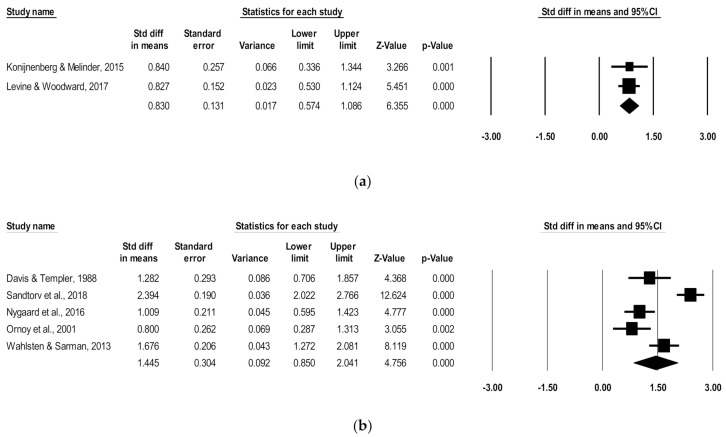
Forest plots comparing combined ADHD subscales of children with POE versus non-exposed children at the (**a**) preschool age and (**b**) school age.

Since ADHD symptoms vary by age, subgroup analyses were run for combined ADHD subscales for studies including a preschool age (4.3–4.5 mean years) and a school age (5.4–11.2 mean years). For the preschool subgroup, two studies were pooled to include 122 children with POE and 134 non-exposed children. There was a significant positive association of POE with reported combined ADHD subscale scores at the preschool age (d = 0.83, 95% CI, 0.57–1.09; *p* < 0.0001), with low heterogeneity (I^2^ = 0), and no poor-quality studies (Figure 3).

For the school-age subgroup, five studies were pooled to include 197 children with POE and 1174 non-exposed children. There was a significant positive association of POE with reported subscale scores at the school age (d = 1.45, 95% CI, 0.85–2.04; *p* < 0.0001), with high heterogeneity (I^2^ = 88.82) (Figure 3). After removing low-quality studies [17,20], the significant positive association persisted in school-aged children (d = 1.57, 95% CI, 0.64–2.50; *p* < 0.0001), with high remaining heterogeneity (I^2^ = 92.3).

**Figure 4:**
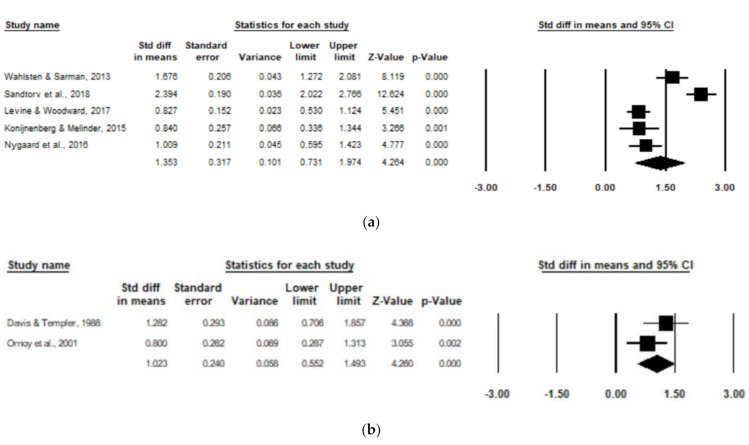
Forest plots comparing combined ADHD subscales of children with POE versus non-exposed children with (**a**) healthy control/comparison groups and (**b**) environmental control/comparison groups.

Subgroup analyses were run for combined ADHD subscales for studies including healthy control/comparison groups (e.g., healthy children from mothers without OUD) and environmental control/comparison groups (e.g., children raised by a father with OUD or children raised in a low SES with environmental deprivation or neglect). For the healthy control/comparison subgroup, five studies were pooled to include 260 children with POE and 1251 non-exposed children. There was a significant positive association of POE with reported combined ADHD subscale scores amongst the healthy control/comparison subgroup (d = 1.35, 95% CI, 0.73–1.97; *p* < 0.0001), with high heterogeneity (I^2^ = 92.19), and one poor-quality study (Figure 4). After removing the low-quality study [17], the significant positive association persisted amongst the healthy control/comparison subgroup (d = 1.27, 95% CI, 0.50–2.04; *p* < 0.0001), with high remaining heterogeneity (I^2^ = 93.8).

For the environmental control/comparison subgroup, two studies were pooled to include 59 children with POE and 60 non-exposed children. There was a significant positive association of POE with reported subscale scores amongst the environmental control/comparison subgroup (d = 1.02, 95% CI, 0.55–1.49; *p* < 0.0001), with low heterogeneity (I^2^ = 33.28), and one poor-quality study [20] (Figure 4).

### 3.7. Evaluation of Bias

Due to an insufficient number of studies, publication bias was not assessed in the combined preschool (*n* = 2), combined environmental control/comparison (*n* = 2), and hyperactivity/impulsivity (*n* = 3) analyses. The Egger test and funnel plot review did not indicate publication bias for reported inattention (SE, 6.92; 95% CI, −42.45–28.61; *p* = 0.49), combined subscale of all children (SE, 5.95; 95% CI, −15.92–14.67; *p* = 0.92), combined subscale of school-aged children (SE, −11.12, 95% CI, 35.40–13.18; *p* = 0.24), or combined subscale of healthy control/comparison group (SE, 10.66; 95% CI, −31.84–36.01; *p* = 0.86) analyses.

## 4. Discussion

This study investigated the association of POE with ADHD symptoms in preschool and school-age children, both independently and combined. In alignment with our hypothesis, findings of this meta-analysis indicated that children with POE have higher hyperactivity/impulsivity, inattention, and combined ADHD symptoms compared to non-exposed controls. This positive association was apparent in children 4–14 years of age. Prenatal opioid exposure had the strongest association with hyperactivity/impulsivity, followed by inattention and then ADHD combined scores, although all effect sizes were considered large [27]. In addition, children with POE appeared to have more ADHD symptoms during school age compared to preschool age. The association of POE and ADHD symptoms seemed to be stronger when compared to healthy control/comparison groups, followed by environmental control/comparison groups.

These findings add to the existing literature suggesting that POE negatively impacts children’s behavioral regulation in preschool and school-age children. While poor behavioral outcomes after POE have been reported [14], findings have been inconsistent or not included studies of older children [13]. Our analysis of data from seven published studies indicated that behavioral dysregulation is evident across childhood. Furthermore, this study adds to existing literature on the specific behavioral challenges of hyperactivity/impulsivity and inattention. While previous meta-analyses have examined behavioral outcomes after POE, none have tested ADHD symptoms specifically. Our study suggests that children with POE are more likely to experience hyperactivity/impulsivity, inattention, and combined symptoms. Moreover, while individual studies have documented elevated hyperactivity [22], impulsivity [21], or inattention [24] after POE, few studies have measured all symptoms of ADHD separately and combined. Thus, the current findings extend the emerging literature on behavioral dysregulation specific to ADHD symptoms in children with POE.

While results of our analyses suggest that ADHD symptoms in children with POE may increase as children age, there are a number of considerations. First, as the preschool subgroup analysis was constrained to two studies, interpretations reflect limited data. Second, all but one included study utilized caregiver report data. The literature suggests that caregivers of children with POE are more likely to have delayed identification of children’s behavioral problems [23]. Thus, it is possible that the higher association with POE and ADHD symptoms at school age (versus preschool age) may be due to delayed identification of ADHD symptoms by caregivers. Third, the origin of this outcome is uncertain as cumulative biological, social, and environmental risk dynamically interact over time.

For instance, Barker’s Fetal Origins of Adult Disease hypothesis states that human fetuses adapt to conditions in utero, which may program or permanently change fetal structure, affecting health over the life-course [41]. Accordingly, opioids may program the developing fetus, which then may interact with environmental and social vulnerabilities at later ages. The increased challenges in time may be related to a more complex and demanding social environment [23]. Longitudinal studies assessing ADHD symptoms indicated that children with POE persisted to have higher ADHD symptoms scores than non-exposed counterparts [16,18,23]. These studies also provided limited clarification on how factors such as maternal polydrug usage, type, dosing, and timing of opioid exposure, NAS diagnosis, change of caregiver, and SES may contribute to outcomes. Researchers speculate any challenges from the POE are further amplified by environmental, social, and biological risk factors [42]. Future studies should continue to investigate these relationships.

Although ADHD symptoms may present differently across developmental stages, their burden on individuals is typically chronic throughout the life-course [43]. Beyond the hallmark symptoms, preschool-aged children with ADHD are likely to have increased conflict with peers and lack of compliance with adults [44]. During school-age years, children with ADHD may experience a surge of oppositional behavior, academic problems, and conflicts with peers [44]. By adolescence, a rise in conflict with parents and emergence of high-risk behaviors are observed higher than the general population [44]. Furthermore, two-thirds of children with ADHD have a comorbid condition [45]. Thus, children with ADHD face greater adversity and stress compared to peers without ADHD [46]. If ADHD persists into adulthood, both personal and professional life may be disrupted [47]. Individuals with ADHD achieve lower educational levels, have higher unemployment rates, and are more likely to experience social relationship challenges than those without ADHD [40,47,48].

Children with POE appeared to have more ADHD symptoms when compared to healthy controls instead of environmental controls, indicating that the postnatal rearing environment impacts the association between POE and ADHD symptoms. While the environmental controls face significant challenges similar to children with POE (e.g., low socio-economic status, father with OUD, environmental deprivation, neglect), the effect size among the environmental subgroup remained large [27], indicating that children with POE represent an exceptionally vulnerable population. This finding should be further explored by future researchers, as there were only two studies in the environmental subgroup analysis. Mothers of children with POE oftentimes have high rates of socio-economic difficulty and comorbid mental health challenges [12]. Many children with POE are at risk of child welfare concerns, with an average of one to two caregiver changes [11]. Children with POE have been removed from parental custody due to maternal substance abuse and mental health issues, child neglect, and maternal imprisonment and physical abuse [11], indicating several adverse childhood experiences. It is well documented that adverse childhood experiences have lasting impacts, including increased mental health problems, risky behaviors, infectious and chronic disease, while lowering education, occupation, and income opportunities [49,50,51].

Findings from this study indicate that children with POE are at high risk for ADHD symptoms that persist throughout childhood [15,17,20,21,23,31,32]. Results suggest that children with POE may benefit from long-term assistance, such as enhanced awareness and surveillance. Future researchers should continue to study long-term behavioral dysregulation after POE. When possible, studies should utilize gold-standard measurements to assess POE, such as urine analysis throughout pregnancy and meconium analysis after birth, as these methods for detecting in utero drug exposure are inexpensive, noninvasive, and will enhance understanding of exposure and outcome relationships [52]. Longitudinal studies should be carefully designed to properly assess biological, social, and environmental risk factors and report outcome data at each time point to increase the availability of aggregate analyses. Furthermore, researchers should report outcomes and subscale data of rating scales controlling for a host of demographic factors and health-related measures to adequately assess the phenotype of children with POE in relation to ADHD symptoms. Lastly, due to variability in behavioral rating scales, researchers investigating ADHD symptoms should carefully review the literature to ensure rating scales match the psychometric properties of the DSM-IV ADHD diagnosis [53].

Strengths of this study include adhering to the recommended protocols related to meta-analyses, including independent reviewers, and achieving a good Cohen’s κ after title and abstract selection and full text retrieval stage. This meta-analysis explored symptoms of hyperactivity/impulsivity and inattention separately as well as combined, adding to the literature on the phenotype of children with POE and specific symptoms of ADHD. Regardless of variability of POE and rating scales, this study encompassed the breadth of available data and found consistent results. Moreover, our study investigated the long-term risk of POE and ADHD symptoms by including subgroup analyses of preschool and school-age children. Lastly, there were no indications of publication bias in any analyses, and sensitivity analyses were conducted, excluding low-quality studies.

While we contacted all primary authors for data, we had no responses with the requested data and were unable to include four pertinent studies for analyses [21,22,24,54]. Nonetheless, the studies excluded were conducted nearly thirty years ago [22], contained low sample sizes [21,22,54], or utilized a cross-sectional study design [22,54]. Thus, it is unlikely that effect sizes would have changed significantly as a result of the missing studies. All outcome data were self-reported from caregiver or teachers; however, while an ADHD diagnosis would be favorable to assess, behavioral scales are commonly used to assess ADHD in the literature [40,55]. Data extracted on biological and social risk were insufficient for further subgroup analyses, which may have explained the significant heterogeneity in analyses. Nonetheless, we attempted to account for this by using a random-effects meta-analysis. Two studies included were historical in nature [20,21]; however, the quality was ranked low only in one [20] of which sensitivity analyses were conducted, excluding low-quality studies. Lastly, the meta-analysis was restricted to English language upon review, which led to the exclusion of only one study that did not report the data needed for analysis.

## 5. Conclusions

This meta-analysis indicates that POE is significantly associated with ADHD symptoms across childhood. Results suggest an increased risk of ADHD symptoms during school age. These findings indicate children with POE may benefit from long-term assistance throughout childhood. Future research is needed to clarify the relationship between biological, environmental, social risk, and ADHD symptoms in children with POE. 

## Figures and Tables

**Table 1:** Descriptive characteristics of (a) hyperactivity/impulsivity and (b) inattention outcomes.

(a) Hyperactivity/Impulsivity												
			POE Children							Non-Exposed Children		
Study, Place of Birth	Measure * Subscale (Reporter)	NAS (%)	Polydrug Exposure	SES	Out Home (%)	POE	Age at Test M (SD)	Outcome M (SD)	*n*	Age at Test M (SD)	Outcome M (SD)	*n*
Konijnenberg, 2015 [32] Norway	BRIEF-P * Inhibit (CG)	50	Tobacco, alcohol, marijuana, amphetamine, benzodiazepine, opioids, MTD, BUP	Yes	14.3	MTD/BUP	4.4 (0.1)	55.97 (11.1)	35	4.3 (0.1)	47.42 (7.1)	31
Davis, 1988 [21] United States	Burk’s * Impulse Control (CG)	N/A	Combination of narcotics	No	N/A	Heroin/MTD	8.5 (2.5)	14.71 (7.9)	28	11.2 (3.0)	8.43 (3.7)	28
Sandtorv, 2018 [31] Norway	SNAP- IV * Hyper/ impulsivity (CG)	44	Number of illicit drugs	NR	NR	OMT	10.4 (2.2)	8.86 (5.3)	57	10.3 (2.0)	1.42 (2.4)	171
**(b) Inattention**												
Konijnenberg, 2015 [32] Norway	BRIEF-P * Working Memory (CG)	50	Tobacco, alcohol, marijuana, amphetamine, benzodiazepine, opioids, MTD, BUP	Yes	14.3	MTD/BUP	4.4 (0.1)	57.46 (11.4)	35	4.3 (0.1)	49.13 (9.9)	31
Davis, 1988 [21] United States	Burk’s * Attention (CG)	N/A	Combination of narcotics	No	N/A	Heroin/MTD	8.5 (2.5)	15.18 (6.3)	28	11.2 (3.0)	8.0 (2.5)	28
Sandtorv, 2018 [31] Norway	SNAP- IV * Inattention (CG)	44	Number of illicit drugs	NR	NR	OMT	10.4 (2.2)	10.79 (4.6)	57	10.3 (2.0)	2.72 (3.5)	171
Nygaard, 2016 [23] Norway	CBCL * Attention (CG)	79	Tobacco, opiates, cannabis, amphetamines, benzodiazepines, alcohol, antipsychotic	Yes	72	Heroin	8.6 (0.6)	5.1 (4.2)	57	8.7 (0.5)	1.7 (4.9)	47

Abbreviations: * = Subscale of measure, BRIEF-P = Behavior Rating Inventory of Executive Function Preschool version, Burk’s = Burk’s Behavior Rating Scales, SNAP IV = Swanson Nolan and Pelham Questionnaire, CBCL 4–18 = Achenbach Child Behavior Checklist, CG = caregiver report, NR = not reported, SES = significant difference between prenatal opioid exposure (POE) and non-exposed group, MTD = methadone, BUP = buprenorphine, OMT = opioid maintenance therapy.

**Table 2:** Descriptive characteristics of combined attention-deficit hyperactivity disorder (ADHD) subscale outcomes.

				POE Children						Non-Exposed Children		
Study, Place of Birth	Measure (Reporter)	NAS (%)	Polydrug Exposure	SES	Out Home (%)	POE	Age at Test M (SD)	Outcome M (SD)	*n*	Age at Test M (SD)	Outcome M (SD)	*n*
Konijnenberg, 2015 [32] Norway	BRIEF-P (CG)	50	Tobacco, alcohol, marijuana, amphetamine, benzodiazepine, opioids, MTD, BUP	Yes	14.3	MTD/ BUP	4.4 (0.1)	113.43 (22.4)	35	4.3 (0.1)	96.55 (17.1)	31
Davis, 1988 [21] United States	Burk’s (CG)	NR	Combination of narcotics	No	N/A	Heroin/MTD	8.5 (2.5)	29.9 (13.5)	28	11.2 (3.0)	16.43 (6.2)	28
Sandtorv, 2018 [31] Norway	SNAP- IV (CG)	44	Number of illicit drugs	NR	NR	OMT	10.4 (2.2)	19.65 (9.1)	57	10.3 (2.0)	4.13 (5.4)	171
Nygaard, 2016 [23] Norway	ADHD Rating Scale (CG)	79	Tobacco, opiates, cannabis, amphetamines, benzodiazepines, alcohol, antipsychotic	Yes	72	Heroin	8.6 (0.6)	15.0 (11.4)	56	8.7 (0.5)	5.8 (5.1)	46
Ornoy, 2001 [20] Israel	Conner’s (CG)	NR	Benzodiazepines, tobacco	No	N/A	Heroin/MTD	8.5 (3.5)	19.89 (10.1)	31	8.5 (3.5)	11.57 (10.7)	32
Levine, 2017 [15] New Zealand	SDQ (CG)	88.2	Nicotine, cannabis, benzodiazepine, other opioid, stimulant, antidepressant, alcohol	Yes	20.6	MTD	4.5 (NR)	4.29 (2.4)	87	4.5 (NR)	2.44 (2.1)	103
Wahlsten, 2013 [17] Sweden	SDQ (TR)	17.8	Relapse indicated, substance not mentioned	NA	32	BUP	5.4 (0.6)	6.7 (2.7)	25	7.5 (1.1)	2.5 (2.5)	900

Abbreviations: BRIEF-P = Behavior Rating Inventory of Executive Function Preschool version, Burk’s = Burk’s Behavior Rating Scales, Conner’s = Conner’s Rating Scales for ADHD, SDQ = Strengths and Difficulties Questionnaire, SNAP IV = Swanson Nolan and Pelham Questionnaire, CG = caregiver report, TR = teacher report, NR = not reported, SES = significant difference between POE and NE group, MTD = methadone, BUP = buprenorphine, OMT = opioid maintenance therapy. Wahlsten 2013 comparison group is from SDQ Sweden normative data [33].

**Table 3:** Quality assessment of included studies: Newcastle–Ottawa Scale.

Cohort	Selection	Comparability	Outcome	Total
Nygaard, E., Slinning, K., Moe, V., and Walhovd, K. B., 2016 [23]	3	2	2	7
Ornoy, A., Segal, J., Bar-Hamburger, R., and Greenbaum, C., 2001 [20]	2	0	1	3
Levine, T. A., and Woodward, L. J., 2017 [15]	4	0	3	7
**Case Control**	**Selection**	**Comparability**	**Exposure**	**Total**
Konijnenberg, C., and Melinder, A., 2015 [32]	2	2	1	5
Davis, D. D., and Templer, D. I.,1988 [21]	3	1	2	6
Sandtorv et al., 2018 [31]	2	2	0	4
Wahlsten and Sarman, 2013 [17]	2	0	0	2

## Data Availability

The data presented in this study are openly available in the published manuscripts cited within this study.

## Appendix A

**Table A1:** PRISMA Checklist.

Section/Topic	#	Checklist Item	Reported on Page #
TITLE			
Title	1	Identify the report as a systematic review, meta-analysis, or both.	1
ABSTRACT			
Structured summary	2	Provide a structured summary including, as applicable: background; objectives; data sources; study eligibility criteria, participants, and interventions; study appraisal and synthesis methods; results; limitations; conclusions and implications of key findings; systematic review registration number.	1
INTRODUCTION			
Rationale	3	Describe the rationale for the review in the context of what is already known.	1–2
Objectives	4	Provide an explicit statement of questions being addressed with reference to participants, interventions, comparisons, outcomes, and study design (PICOS).	2–3
METHODS			
Protocol and registration	5	Indicate if a review protocol exists, if and where it can be accessed (e.g., Web address), and, if available, provide registration information including registration number.	2
Eligibility criteria	6	Specify study characteristics (e.g., PICOS, length of follow-up) and report characteristics (e.g., years considered, language, publication status) used as criteria for eligibility, giving rationale.	2–3
Information sources	7	Describe all information sources (e.g., databases with dates of coverage, contact with study authors to identify additional studies) in the search and date last searched.	3
Search	8	Present full electronic search strategy for at least one database, including any limits used, such that it could be repeated.	3
Study selection	9	State the process for selecting studies (i.e., screening, eligibility, included in systematic review, and, if applicable, included in the meta-analysis).	2–5
Data collection process	10	Describe method of data extraction from reports (e.g., piloted forms, independently, in duplicate) and any processes for obtaining and confirming data from investigators.	2–5
Data items	11	List and define all variables for which data were sought (e.g., PICOS, funding sources) and any assumptions and simplifications made.	3
Risk of bias in individual studies	12	Describe methods used for assessing risk of bias of individual studies (including specification of whether this was done at the study or outcome level), and how this information is to be used in any data synthesis.	3
Summary measures	13	State the principal summary measures (e.g., risk ratio, difference in means).	3
Synthesis of results	14	Describe the methods of handling data and combining results of studies, if done, including measures of consistency (e.g., I^2^) for each meta-analysis.	3
Risk of bias across studies	15	Specify any assessment of risk of bias that may affect the cumulative evidence (e.g., publication bias, selective reporting within studies).	3
Additional analyses	16	Describe methods of additional analyses (e.g., sensitivity or subgroup analyses, meta-regression), if done, indicating which were pre-specified.	3
RESULTS			
Study selection	17	Give numbers of studies screened, assessed for eligibility, and included in the review, with reasons for exclusions at each stage, ideally with a flow diagram.	3–4
Study characteristics	18	For each study, present characteristics for which data were extracted (e.g., study size, PICOS, follow-up period) and provide the citations.	5–7
Risk of bias within studies	19	Present data on risk of bias of each study and, if available, any outcome level assessment (see item 12).	8–11
Results of individual studies	20	For all outcomes considered (benefits or harms), present, for each study: (a) simple summary data for each intervention group (b) effect estimates and confidence intervals, ideally with a forest plot.	8–11
Synthesis of results	21	Present results of each meta-analysis done, including confidence intervals and measures of consistency.	8–11
Risk of bias across studies	22	Present results of any assessment of risk of bias across studies (see Item 15).	8–11
Additional analysis	23	Give results of additional analyses, if done (e.g., sensitivity or subgroup analyses, meta-regression [see Item 16]).	8–11
DISCUSSION			
Summary of evidence	24	Summarize the main findings including the strength of evidence for each main outcome; consider their relevance to key groups (e.g., healthcare providers, users, and policy makers).	11–13
Limitations	25	Discuss limitations at study and outcome level (e.g., risk of bias), and at review level (e.g., incomplete retrieval of identified research, reporting bias).	13
Conclusions	26	Provide a general interpretation of the results in the context of other evidence and implications for future research.	13–14
FUNDING			
Funding	27	Describe sources of funding for the systematic review and other support (e.g., supply of data); role of funders for the systematic review.	NA
